# Optimization of EPS Production and Characterization by a Halophilic Bacterium, *Kocuria rosea* ZJUQH from Chaka Salt Lake with Response Surface Methodology

**DOI:** 10.3390/molecules22050814

**Published:** 2017-05-16

**Authors:** Di Gu, Yingchun Jiao, Jianan Wu, Zhengjie Liu, Qihe Chen

**Affiliations:** 1 Department of Food Science and Nutrition, Zhejiang University, Hangzhou 310058, China; 3140100583@zju.edu.cn (D.G.); hangzhouwjn@163.com (J.W.); liuzjemail@163.com (Z.L.); 2 College of Eco-Environmental Engineering, Qinghai Province, Xining 810016, China; jyc_22@163.com

**Keywords:** halophilic bacterium, *Kocuria rosea* ZJUQH, exopolysaccharides, optimization, response surface methodology

## Abstract

With the rising awareness of microbial exopolysaccharides (EPSs) application in various fields, halophilic microorganisms which produce EPSs have received broad attention. A newly identified *Kocuria rosea* ZJUQH CCTCC M2016754 was determined to be a moderate halobacterium on account of its successful adaption to the environment containing 10% NaCl. The optimal combination of fermentation medium compositions on EPS production was studied. In this work, a fractional factorial design was adopted to investigate the significant factors that affected EPS production. The factors of KCl and MgSO_4_ were found to have a profound impact on EPS production. We utilized central composite design and response surface methodology to derive a statistical model for optimizing the submerged culture medium composition. Judging from these experimental results, the optimum culture medium for producing EPSs was composed of 0.50% casein hydrolysate, 1.00% sodium citrate, 0.30% yeast extract, 0.50% KCl, 0.50% peptone, and 5.80% MgSO_4_ (initial pH 7.0). The maximal EPS was 48.01 g/L, which is close to the predicted value (50.39 g/L). In the validation experiment, the highest concentration of 70.64 g/L EPSs was obtained after 120 h under the optimized culture medium in a 5-L bioreactor. EPS from this bacterium was also characterized by differential scanning calorimetry (DSC) and Fourier transform infrared analysis (FT-IR). The findings in this study imply that *Kocuria rosea* ZJUQH has great potential to be exploited as a source of EPSs utilized in food, the pharmaceutical and agriculture industry, and in the biotreatment of hypersaline environments.

## 1. Introduction

Halophilic microorganisms are capable of growing and carrying out their metabolism under hypersaline conditions. As a result of adaptation to their environment, they have evolved various characters and salt acclimation strategies. Halophilic microorganisms can be classified according to the salt concentrations that they need to grow in, as slight halophiles (2–5% NaCl), moderate halophiles (5–20% NaCl), and extreme halophiles (20–30% NaCl) [1]. Extracellular polysaccharides (exopolysaccharides, EPSs) are one of the most important group of substances produced by microorganisms for their survival in hypertonic environment [2]. They occur in two forms: as a capsule closely associated with the cell surface or as slime polysaccharides loosely associated with the cell surface [3]. Those polysaccharides could also be differentiated by their chemical composition. Homopolysaccharides (HoPS) are composed of only one monosaccharide (glucose or fructose, mainly), and heteropolysaccharides (HePS) comprise repeating units of different monosaccharides [4]. Other residues such as sn-glycerol-3-phosphate, N-acetyl-amino sugars, phosphate, and acetyl groups can also be found [5]. EPSs have important ecological and physiological functions and play special roles in protecting the microorganisms that produce them. They are believed to protect cells against antimicrobial substances, desiccation, bacteriophages, osmotic stress, and antibodies as well as to permit adhesion to solid surfaces and biofilm formation [6,7,8]. Microbial polysaccharides have found a broad spectrum of applications in industry. In the food industry, these polymers are used as biothickeners to improve food quality and texture because of their stabilizing, gelling or emulsifying properties [9]. As supplemented with dextran, superior structural and textural extrudate characteristics were achieved in high dietary fiber extrusion [10]. In the pharmaceutical industry, polysaccharides can be used as a hydrophilic matrix for controlled release of drugs [9], as an anti HIV agent [11], and to enhance nonspecific immunity [12]. In the agriculture sector, the fluidity of fungicides, herbicides, and insecticides has been improved by the addition of xanthan, which results in the uniform suspension of solid components in formulations [13]. In addition, their functional properties also include bioflocculants [14], biosorption of heavy metals [15], and chemical products [16,17]. With the industrial development, fresh water and hypersaline environments are frequently contaminated with organic pollutants [18]. At present, the halophiles show great potential in bioremediation of those organic compounds in hypersaline conditions [19].

In this study, an endophyte halobacterium *Kocuria rosea* ZJUQH that was previously isolated from Chaka Salt Lake, Tibet was found to produce EPSs during liquid submerged cultivation. This study was done on its fermentation character, defining the most significant components in the medium makeup, and then investigating the optimal fermentation medium components for higher EPS production.

## 2. Results

### 2.1. Determination of the Salt Tolerance

**Figure 1:**
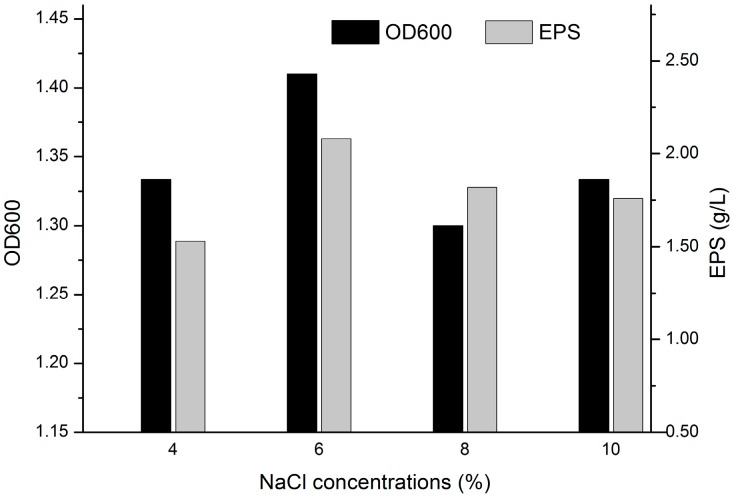
Determination of the salt tolerance by *K. rosea* ZJUQH. Different amounts of NaCl at 4%, 6%, 8%, 10% (*w*/*v*) were used independently in the fermentation media. The culture flasks were inoculated with 5% (*v*/*v*) culture seed in cell growth of 48 h and then incubated on a 130 rpm rotary shaker at 25 °C, for 120 h. Then OD600 and exopolysaccharide (EPS) formation were measured.

We previously found that the isolated bacterium showed better salt tolerance on the basis of its origin which is a salt lake with very high salinity. The correlation between the salt tolerance and EPS formation is still needed to be made clear. As shown in Figure 1, *K. rosea* ZJUQH is able to survive in the culture environment containing up to 10% NaCl. Thus, it was considered to be a moderate halophile. Based on the record of cell biomass and EPS production, the maximum cell biomass (2.08 g/L) was obtained with 6% NaCl. Moreover, it was found that cell growth was in accordance with the EPS formation.

### 2.2. Screening of the Fermentation Culture Media

**Figure 2:**
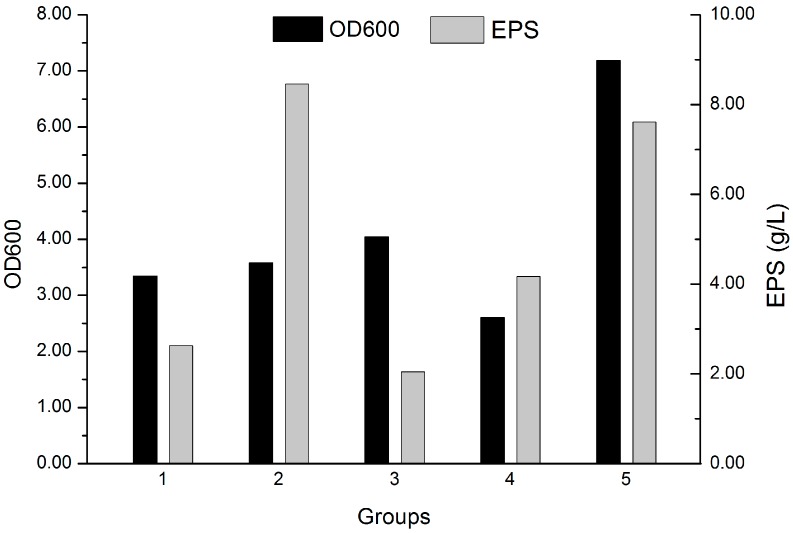
Screening of the fermentation culture media. The culture flasks were inoculated with 5% (*v*/*v*) culture seed in cell growth of 48 h and then incubated on a 130 rpm rotary shaker at 25 °C, for 120 h. The constituents of fermentation culture are described in Table 1. Then samples were checked for cell biomass and EPS formation.

Formulae of five different fermentation culture media are shown in Table 1. M2 had rather abundant glucose as carbon source. While there was starch instead in M4 with less salt. M1, M3, M5 were rich in nitrogen source. Peptone and beef extract were used in M1 and M3 while casein hydrolysate in M5. Moreover, M1 and M5 had relatively high salinity. Based on cell biomass and EPS formation variations presented in Figure 2, it was found that the culture medium M5 was considered to be the optimal fermentation culture for both EPS production and cell accumulation. Although fermentation medium M2 is better for EPS formation, it showed little beneficial effect on cell growth. As compared with these two culture media compositions, it is clear that a low ratio of C/N in the fermentation medium is useful for EPS production.

### 2.3. Optimization of Culture Media Compositions for EPS Production

#### 2.3.1. Screening Stage: Half Fractional Factorial Design

The screening experiments were designed to evaluate the impacts of six factors which were concentrations of casein hydrolysate, sodium citrate, yeast extract, KCl, peptone, and MgSO_4_. A two-level half fractional factorial design was employed and the result of the fractional factorial design is shown in Table 2 and Table 3. The production of EPSs referred to as g/L varied markedly in a range from 12.34 g/L to 44.77 g/L. The lowest production of EPSs was obtained when minimal levels of KCl and MgSO_4_ were used. Clearly, these variables significantly affected the production of EPSs. As can be seen from Table 4, the factors of KCl and MgSO_4_ were found to be significant at the probability level of 99% for EPS production, which demonstrated that the two factors had significant effects on the formation of EPS. The main effects of these variables were positive. Other factors demonstrated an insignificant effect on the production of EPS. The effects of casein hydrolysate, sodium citrate, yeast extract, KCl, peptone, and MgSO_4_ on accumulation of EPSs were also analyzed by multiple regression techniques. However, based on the results of the *F*-test for variance between the averages of observation of two-level experiments and the center point it was shown that the difference was not significant (*p* > 0.05) (Table 3). These results indicated that the optimum point was not in the domain of our experiment. Thus, we adopted the experiment of steepest ascent path to reach the optimum domain. KCl (x_4_) and MgSO_4_ (x_6_) were then determined to be the main factors for further optimization.

#### 2.3.2. The Steepest Ascent Experiment

The path began at the center point of the RSM design space, serving as the origin for the steepest ascent experiment, and stretched outside the original design space to explore the outside region. A sequence of equally spaced steps along the path was selected which formed a set of experimental runs consisting of different concentrations of KCl and MgSO_4_.

The steepest ascent is a method which uses the magnitude and sign of linear effects to make sure of the direction toward predictive higher responses. The path begins at the center of the current design space and stretches well outside the original design space to explore the outside region. A sequence of equally spaced steps along the path were then selected which formed a set of experimental runs consisting of different KCl and MgSO_4_ concentrations [20]. Thus, KCl (x_4_) and MgSO4 (x_6_) concentrations were increased in order to improve the formation of EPS (Y_1_). Other factors (x_1_, x_2_, x_3_, x_5_) were fixed at zero level. The values of EPS production obtained in these experiments are summarized in Table 5. It can be seen that the data fell within our expectations with the increase of EPS values. The highest yield of EPS (63.13 g/L) was achieved in the last step, yet this value may not have approached the maximum point.

#### 2.3.3. Optimization of KCl and MgSO_4_ for the Maximal Production of EPSs

Response surface methodology using central composite design (CCD) was employed to determine the optimal levels of two selected factors (KCl and MgSO_4_) that influenced EPS production. Table 6 shows the experimental CCD and its corresponding results. Regression analysis was performed to fit the response function with the experimental data. The statistical significance of the second-order model equation was checked by an *F*-test (ANOVA) with data shown in Table 7. The fit value, termed R^2^ (determinant coefficient), of the polynomial model was calculated to be 0.94, indicating that 94% of the variability in the response could be explained by the second-order polynomial prediction equation given below The ANOVA results showed that this model is appropriate. It also suggested that the production of polysaccharide by a *K. rosea* ZJUQH was primarily determined by the linear and quadratic terms of KCl (x_4_) and MgSO_4_ (x_6_) concentrations of the model and no significant interaction existed between the two factors. The equation for the EPSs showed positive linear effect and negative quadratic effect:

Y = 63.05 + 0.32 × x_1_ + 11.01 × x_2_ + 1.48 × x_12_ − 2.08 × x_22_ + 1.36 × x_1_ × x_2_

**Figure 3:**
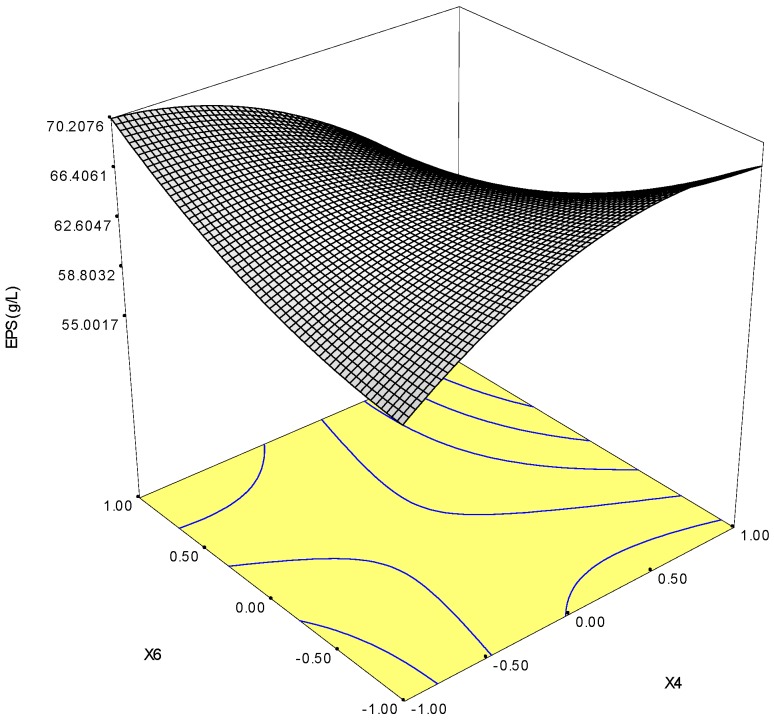
Response surface plot of KCl (x_4_) and MgSO_4_ (x_6_) against EPS production by *K. rosea* ZJUQH.

The three-dimensional plot obtained from the calculated response surface is shown in Figure 3, Three-dimensional response surface plots of KCl (x_4_) and MgSO_4_ (x_6_) against polysaccharide production (Y_1_) can explain the results of the statistical and mathematical analyses. It is evident from the plot that Y reached its maximum at a combination of coded level −1 (x_4_), +1 (x_6_). The predicted maximum production of EPS is 70.25 g/L when KCl (x_4_) is 5.00 g/L, and MgSO_4_ (x_6_) 58.00 g/L. This theoretical maximum value is 32.25% higher than the yield from the control fermentation without the optimization process (13.15 ± 0.72 g/L).

### 2.4. Validation of the Optimized Culture Conditions

**Figure 4:**
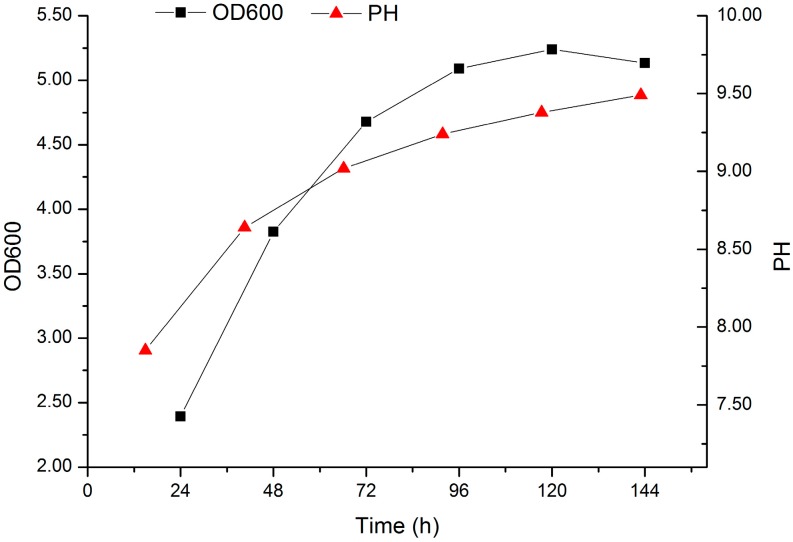
Time-course process of batch fermentation in a 5-L bioreactor for EPS production by a *K. rosea* ZJUQH under optimized condition. The optimized fermentation medium contained 5 g/L casein hydrolysate, 10 g/L sodium citrate, 3 g/L yeast extract, 5 g/L KCl, 5 g/L peptone, 58 g/L MgSO_4,_ and the initial pH was adjusted to 7.0. Agitation speed 150 r/min, aeration rate 1.2 vvm, 25 °C.

In order to validate the adequacy of this model, verification experiments were carried out at the predicted optimal culture media. The mean concentration of the obtained EPSs from triplicate trials in shake flasks was 72.01 ± 1.54 g/L, which was close to the predicted value. Furthermore, the suitability of this model was investigated by batch fermentation of EPSs using a *K. rosea* ZJUQH in a 5-L bioreactor. The pH, cell growth and the production of EPSs were monitored during the submerged cultivation (Figure 4). As shown in Figure 4, the highest concentration of 72.01 g/L EPSs was obtained at 144 h cultivation, followed by 70.64 g/L at 120 h while the OD600 achieved a maximum at 120 h and then dropped. In addition, the pH kept rising during the procedure of fermentation. 

### 2.5. Effects of KCl and MgSO_4_ on EPS Production

**Figure 5:**
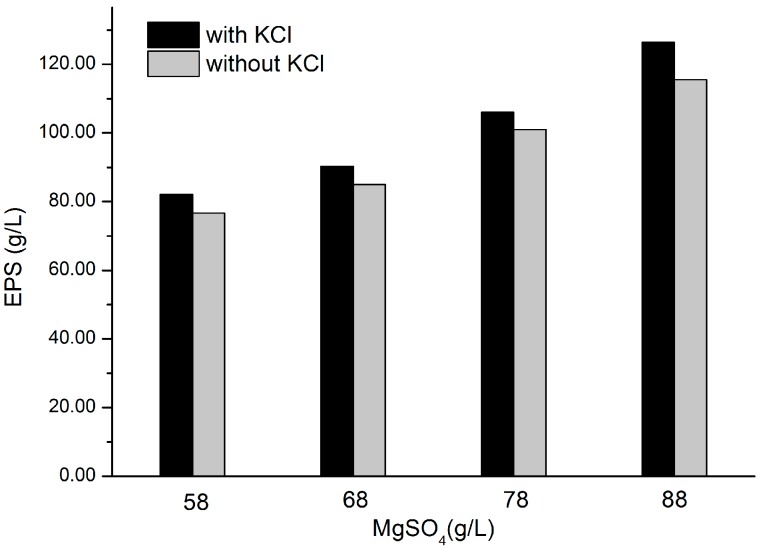
Effects of KCl and MgSO_4_ on EPS production. Different amounts of MgSO_4_ at 5.8%, 6.8%, 7.8%, 8.8% (*w*/*v*) and a constant concentration of KCl were used independently in the fermentation media as the experimental group. No KCl was added into the blank control but a corresponding concentration of MgSO_4_. The culture flasks were inoculated with 5% (*v*/*v*) culture seed in cell growth of 48 h and then incubated on a 130 rpm rotary shaker at 25 °C, for 120 h. The liquid fermentation medium contained 5 g/L casein hydrolysate, 10 g/L sodium citrate, 3 g/L yeast extract, 5 g/L peptone, and the initial pH was adjusted to 7.0.

As we can see in Figure 5, cell growth and EPS formation are promoted due to the existence of KCl. As the concentration of KCl remains constant, the addition of MgSO_4_ could induce the formation of EPS. More importantly, this bacterium can tolerate a high level of MgSO_4_. We reasoned that both KCl and MgSO_4_ are of great importance in EPS production. As previously reported, relative abundances of haloquadratum-related sequences and concentrations of potassium, magnesium and sulfate have a positive correlation [21]. Ionic ratios also affect the efficiency of cellular ion pumps and antiporters which are applied to balance intracellular osmolarity and establish electrochemical gradients for energy production and nutrient transport [22]. The detailed mechanism underlined will be elucidated in the future when more proof has been provided.

### 2.6. Validation of the Essentiality of MgSO_4_

**Figure 6:**
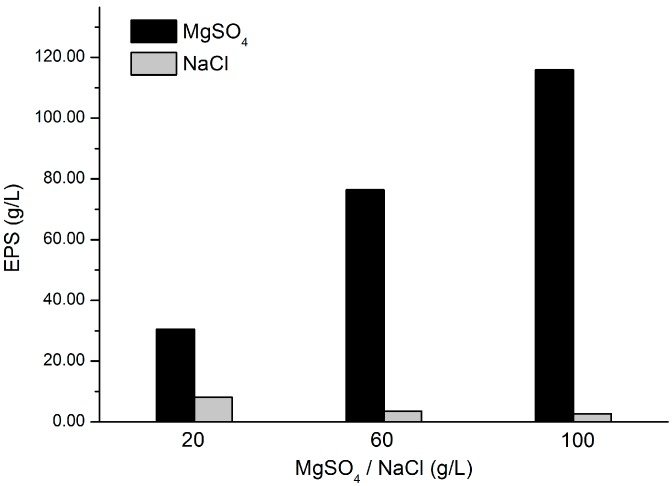
Validation of the essentiality of MgSO_4_. Different concentrations of MgSO_4_ at 2%, 6%, 10% (*w*/*v*) were used independently in the fermentation media and in the blank control, an equal amount of NaCl took the place of MgSO_4_. MgSO_4_ in the seed culture medium was also replaced by NaCl with the same concentrations. The culture flasks were inoculated with 5% (*v*/*v*) culture seed in cell growth of 48 h and then incubated as described before. Samples were assayed for EPS formation after 120 h cultivation.

After we found the importance of MgSO_4_ in EPS production in the previous experiments, we tried to replace MgSO_4_ with an equal amount of NaCl in order to see if it is an alternative. On the basis of Figure 6, the disparity of EPS production was obvious when comparing every two cultures of the same concentration of MgSO_4_ and NaCl. The environment without MgSO_4_ was too adverse for *K. rosea* ZJUQH to produce EPSs. This implied that MgSO_4_ was of great importance and irreplaceable.

### 2.7. TEM Observation

**Figure 7:**
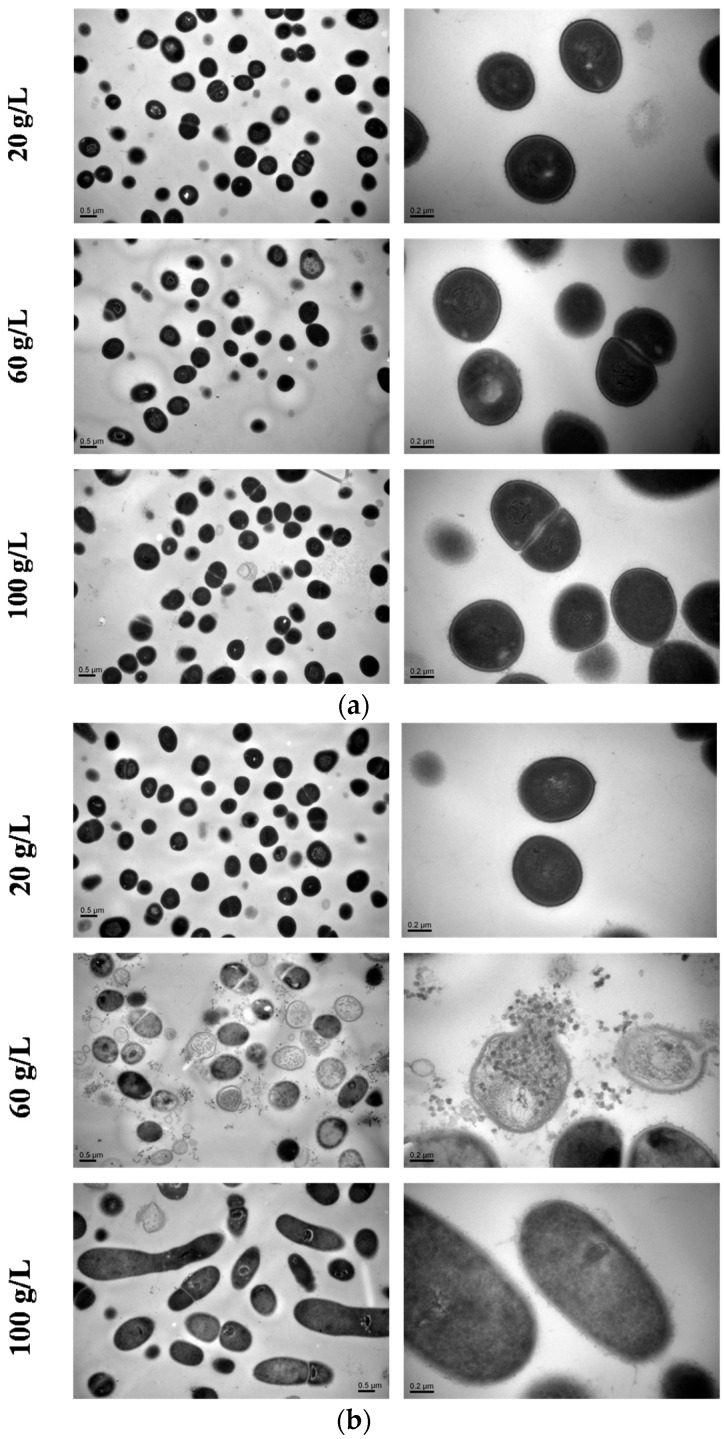
Comparison of phenotypic properties in *K. rosea* ZJUQH cells with different amounts of MgSO_4_ or NaCl. (**a**) Transmission electron microscopy (TEM) observation of 20, 60, 100 g/L MgSO_4_ treatment; (**b**) TEM observation of 20, 60, 100 g/L NaCl treatment.

Presented in Figure 7, the cells in the lowest salinity were mostly stable regular spheres, which exhibited an intact and thick cell wall. With the increasing salinity of the fermentation culture, the cell wall became thinner and more fragile. Intracellular organelles were disintegrated indicating that the cells had become vulnerable. Note that the cell division phenomenon occurred more frequently with high salinity. Comparing Figure 7b with Figure 7a, it can be seen that cell wall rupture or cell collapse of *K. rosea* ZJUQH was much severer in the case of NaCl, especially in 60 g/L NaCl. As for higher concentration of NaCl, there was a great change in cellular morphology. Cells became longer or ellipsoidal, which may itself be an efficient salinity acclimation strategy.

### 2.8. Characterization of EPS

#### 2.8.1. Molecular Mass Determination and Analysis of Monosaccharide Composition

The molecular weight of purified EPS was determined by the SEC-MALLS-RI system. The average molecular mass of purified EPS was estimated to be 5.659 × 10^4^ Da.

**Figure 8:**
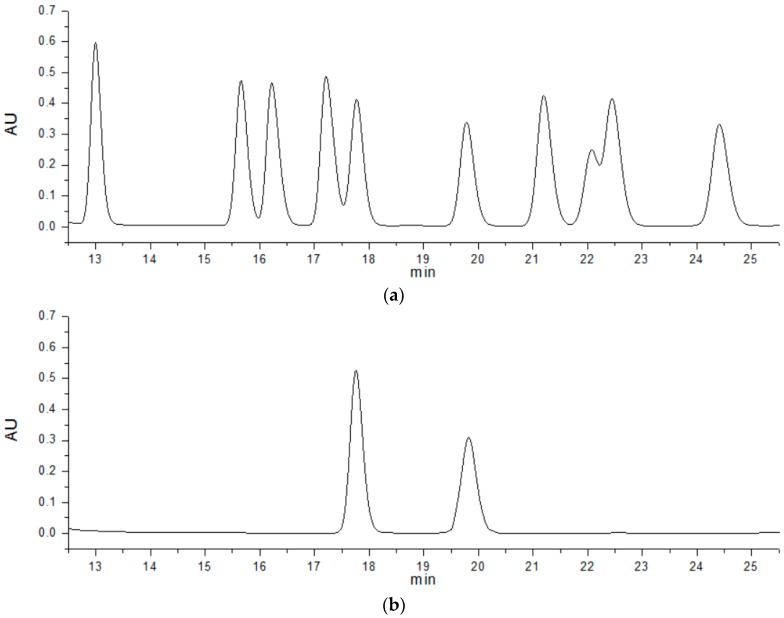
Monosaccharide compositions of the purified EPS (**a**) standard substance; (**b**) purified EPS.

The monosaccharide composition of the polysaccharides was analyzed by a 1-phenyl-3-methyl-5-pyrazolone high performance liquid chromatography (PMP-HPLC) method. The results of HPLC analysis are represented in Figure 8. The peaks of standard monosaccharide in Figure 8a from left to right are mannose (Man), rhamnose (Rha), glucuronic acid (GluA), galacturonic acid (GalA), galactose (Gal), glucose (Glu), xylose (Xyl), arabinose (Ara), and fucose (Fuc). As we can see in Figure 8b, the first peak represents an internal standard and the latter glucose. Thus, the purified EPS was glucan consisting of only glucose.

#### 2.8.2. Determination of Total Sugar, Uronic Sugar and Protein

The total sugar content of purified EPS was 85.86%. According to the results of the PMP-HPLC method and the Bradford method, the purified EPS contained no uronic sugar and 0.089% protein.

#### 2.8.3. DSC Analysis

**Figure 9:**
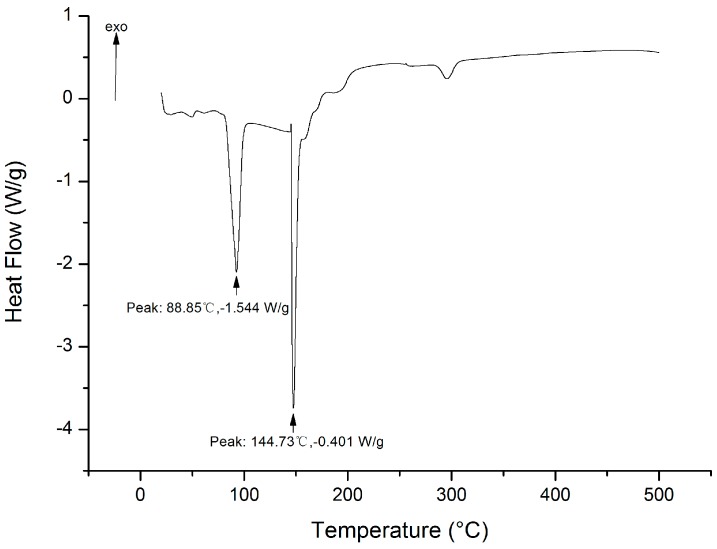
Differential scanning calorimetry (DSC) thermograms of EPS.

Differential scanning calorimetry (DSC) measurement was used to evaluate the thermal behavior of the purified EPS. According to Figure 9, the DSC curve showed two endothermic sharp peaks at 88.85 °C and 144.73 °C. The former stood for the loss of adsorbed water in the polymer matrix and the latter indicated a melting point. No exothermic peak was observed, which means there was no oxidization reaction or cross-linking reaction involving EPS during the heating procedure.

#### 2.8.4. FT-IR Analysis

**Figure 10:**
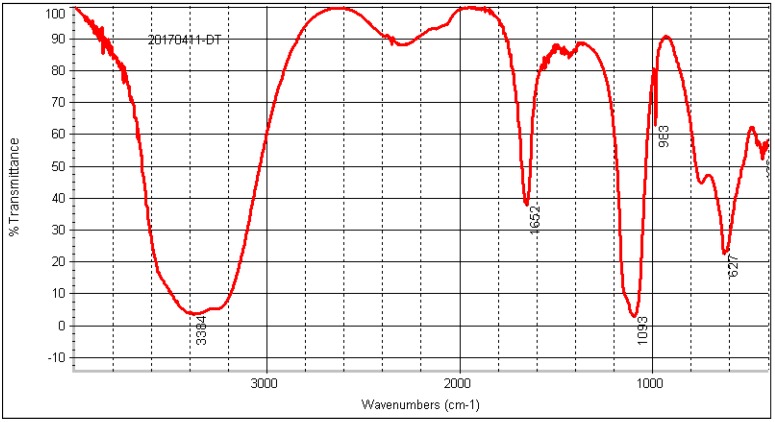
Fourier transform infrared spectrum of EPS.

In order to investigate the structure of EPS in more detail, we used a Fourier-transform infrared spectrophotometer to acquire more information about radical groups in the polymer matrix. An EPS FT-IR spectrum was performed in the region of 4000–400 cm^−1^. As shown in Figure 10, EPS displayed typical peaks of EPSs at 3384, 1652, and 1093 cm^−1^. The broadly-stretched intense peak at 3384 cm^−1^ [23]. The bands at 1652 cm^−1^ could be conformed as stretching vibrations of the carboxylate bonds [24]. The absence of bands in the region of 2600 and 2500 cm^−1^ indicates that EPS does not contain sulfydryl. Moreover, it has been reported that each particular polysaccharide has a specific band in the range 1200–1000 cm^−1^ which is dominated by ring vibrations overlapping with stretching vibrations of the (C-O-H) side groups and the (C-O-C) glycosidic bond vibration [25]. EPS has a particular band at 1093 cm^−1^ indicating a possible α (1→6) glucosidic bond with a certain flexibility.

## 3. Discussion

For halophilic microorganisms in extreme environments, as is reported, there are two general adaptive strategies to achieve salinity and osmotic stress tolerance [26]. The ‘salt in’ strategy: maintaining favorable osmotic pressure by accumulating high cytoplasmic concentrations of potassium ions (K^+^) [27,28]; The ‘organic solutes in’ strategy: using the osmolytes comprised of sugars, amino acids, quaternary amines, polyols [29,30] or ectoines [31,32]. As a kind of ‘organic solutes in’ strategy, exopolysaccharides (EPSs) are polymers produced by many microbes, including fungi, microalgae, bacteria, and archaea [33]. Because of their ecological (colony stimulating), physiological (anti-tumor, interferon induction, anti-viral) and physicochemical (thickening, suspending, stabilizing) properties [34,35], EPSs are widely used in the pharmaceutical industry, the food industry, and the field of agriculture [8,36]. In recent years, EPSs have attracted more attention in their potential application in sewage treatment [19,37], bioflocculant [38], skin care products [39], and food additives [40]. During the past 50 years, a considerable number of EPSs have been studied [41]. However, their properties for applications have not been thoroughly understood because it is often difficult to identify their chemical structures. Thus, little research has been done to establish a correlation between the composition of EPS and their applications [42]. On the basis of existing studies, the properties of polysaccharides are influenced by the location, composition, molecular mass, and conformation or other criteria. EPS characteristics and amounts can be influenced by several factors such as composition of the medium (carbon and nitrogen sources), as well as incubation conditions (temperature, pH, time, etc.) [43,44].

Microbes in an extreme environment such as hypersaline habitats have triggered broad biotechnology interest, which is because of their potential unusual properties. An endophyte halobacterium *K. rosea* ZJUQH isolated from Chaka Salt Lake was investigated in this study, which showed a good performance in salt tolerance and EPS production. After optimization of the fermentation culture medium, its capability of producing EPS was significantly improved. It is worth noting that Mg^2+^ played a key role in both bacterial growth and EPS production which cannot be replaced by NaCl. We also found the necessity of K^+^ in the EPS production process since the increase of only Mg^2+^ content cannot raise the yield dramatically as is the case with an increase of both Mg^2+^ and K^+^ in the fermentation culture. According to the results of this study, it is worth investigating the biosynthetic pathway of EPSs and the role that MgSO_4_ plays in this process. The relation between cellular morphology change and salinity acclimation attracted our interest as well. It can be seen from the transmission electron microscopy images that cells changed shape such as cell wall thinning to adapt to the high salinity environment. With the increasing salinity, cells ruptured when they could not endure the salinity anymore which was more likely to happen in the case of NaCl than MgSO_4_. The structure of EPS as well as the correlation between the composition of EPS and their applications also needs further study. What is more, we are interested in the effect of culture conditions such as temperature, aeration rate, pH on EPS production in our future research. Besides the extracellular sector, more work is required to study the intracellular substances in relation to the osmotic balance of the cells or other potential functions.

## 4. Materials and Methods

### 4.1. Microorganism and Culture Media

*Kocuria rosea* ZJUQH was used in this study. This bacterium was preserved at Zhejiang University and deposited in the China General Microbiological Culture Collection Center (ZJUQH CCTCC M2016754). This strain showed good performance for cell growth and EPS formation in a culture environment containing 10% NaCl, and it was determined to be a moderate halobacterium. The initial slant medium was composed of 5 g/L casein hydrolysate, 10 g/L sodium citrate, 3 g/L yeast extract, 2 g/L potassium chloride, 5 g/L peptone, 10 g/L magnesium sulfate, 20 g/L agar at pH 7.0. The seed culture medium contained 5 g/L casein hydrolysate, 10 g/L sodium citrate, 3 g/L yeast extract, 2 g/L KCl, 5 g/L peptone, and 30 g/L MgSO_4_ (initial pH 7.0). The liquid fermentation medium was comprised of 3–7 g/L casein hydrolysate, 5–15 g/L sodium citrate, 1–5 g/L yeast extract, 0–8 g/L potassium chloride, 3–8 g/L peptone, 10–80 g/L magnesium sulfate and the initial pH was adjusted to 6.8–7.2.

### 4.2. Culture Conditions

A loop full of cells of *K. rosea* ZJUQH from slant culture were inoculated into 100 mL of fresh seed medium in 250 mL flasks and aerobically incubated for 36–60 h at 25 °C, 130 rpm on a rotary shaker. Then, 5% (*v*/*v*) of the seed culture was transferred to 100 mL fermentation medium in 250 mL Erlenmeyer flasks followed by incubation on a 130 rpm rotary shaker at 25 °C, for 120 h. The fermentation media were varied in term of the experimental design. Batch fermentations were studied in a 5-L stirred bioreactor (Biostat B plus, Sartorius Stedim Biotech Inc., Goettingen, Germany) with a 4.0 L working volume. The process set parameters were pH 7.0; temperature, 30 °C; agitation speed, 200 r/min; and aeration rate, 1.2 vvm. Both agitation and aeration were kept constant throughout the entire process.

### 4.3. Determination of Salt Tolerance

The fermentation media containing only NaCl were adopted to determine the salt tolerance of *K. rosea* ZJUQH. Different concentrations of NaCl at 4%, 6%, 8%, 10% (*w*/*v*) were used independently in the fermentation media. The culture flasks were inoculated with 5% (*v*/*v*) culture seed in cell growth of 48 h and then incubated as described before. Samples were checked for cell biomass and EPS formation after 120 h cultivation.

### 4.4. Screening of the Fermentation Culture Compositions

In order to determine the optimal fermentation culture for EPS formation, screening experiments were designed using five different kinds of media formula (Table 1). The culture flasks were inoculated with 5% (*v*/*v*) culture seed in cell growth of 48 h and then incubated as described before. Samples were checked for cell biomass and EPS formation after 120 h cultivation.

### 4.5. Effects of KCl and MgSO_4_ on EPS Production

Different amounts of MgSO_4_ at 5.8%, 6.8%, 7.8%, 8.8% (*w*/*v*) and a constant concentration of KCl were used independently in the fermentation media as the experimental groups. No KCl was added into the blank control and other factors were kept the same. The culture flasks were inoculated with 5% (*v*/*v*) culture seed in cell growth of 48 h and then incubated as described before. Samples were measured for EPS formation after 120 h cultivation.

### 4.6. Validation of the Essentiality of MgSO_4_

To verify the importance of MgSO_4_, we set a contrast experiment. In the experimental group, different concentrations of MgSO_4_ at 2%, 6%, 10% (*w*/*v*) were used independently in the fermentation media. In the blank control, NaCl was substituted for MgSO_4_ with the same concentrations. MgSO_4_ in the seed culture medium was also replaced by an equal amount of NaCl. The culture flasks were inoculated with 5% (*v*/*v*) culture seed in cell growth of 48 h and then incubated as described before. Samples were assayed for EPS formation after 120 h cultivation.

### 4.7. Transmission Electron Microscopy (TEM)

To investigate the impact of MgSO_4_ and NaCl on cell morphology and intracellular structures, we used TEM analysis. The seed culture was conducted as mentioned before. Cells were incubated in the fermentation medium with 20, 60, 100 g/L MgSO_4_ or NaCl on a 130 rpm rotary shaker at 25 °C, for 120 h before TEM observation. 

Sample processing procedures of TEM:

- Double fixation: The specimen was first fixed with 2.5% glutaraldehyde in phosphate buffer (0.1M, pH 7.0) for more than 4 h; washed three times in the phosphate buffer (0.1 M, pH 7.0) for 15 min at each step; then postfixed with 1% OsO4 in phosphate buffer (0.1 M, pH 7.0) for 1–2 h and washed three times in the phosphate buffer (0.1 M, pH 7.0) for 15 min at each step.
- Dehydration: The specimen was first dehydrated by a graded series of ethanol (30%, 50%, 70%, 80%, 90%, 95%, and 100%) for about 15 to 20 min at each step, then transferred to absolute acetone for 20 min.
- Infiltration: The specimen was placed in 1:1 mixture of absolute acetone and final Spurr resin mixture for 1 h at room temperature, then transferred to 1:3 mixture of absolute acetone and the final resin mixture for 3 h and to final Spurr resin mixture for overnight.
- Embedding and ultrathin sectioning: Specimen was placed in an Eppendorf containing Spurr resin and heated at 70 °C for more than 9 h. The specimen was sectioned in LEICA EM UC7 ultratome and sections were stained by uranyl acetate and alkaline lead citrate for 5 to 10 min respectively and observed in a Hitachi Model H-7650 TEM.

### 4.8. Analytical Methods

#### 4.8.1. Determination of Cell Dry Weight and Cell Biomass

At required time intervals, at least three 20 mL fermentation broth were withdrawn aseptically from the respective flask and then harvested by centrifugation. The precipitates were washed with distilled water and dried at 60 °C for 12 h to determine the dry cell weight (DCW). The cell dry weight was then determined gravimetrically and expressed in g/L. The cell free supernatant was used for the recovery of EPS. OD600 was also adopted to reflect the biomass density qualitatively using a spectrophotometer after incubated culture. 

#### 4.8.2. Recovery of Native Crude EPSs and Quantification

The method used for the recovery of crude EPSs from the fermentation broth is as referred to in the reported literature [3]. After fermentation, the culture broth was centrifuged at 3000 rpm for 30 min to separate completely the biomass. A measured volume of the cell free supernatant (10 mL) was added into three volumes of ice-cold alcohol followed by keeping overnight at 4 °C to precipitate EPS. The precipitate formed was then recovered by centrifuging at 3000 rpm for 25 min. The precipitated EPS was then dissolved in 10 mL water on a boiling water bath for 30 min. After centrifuging at 3000 rpm for 15 min, all the EPS was in the supernatant. The supernatant was deproteinized by using the Sevag method [45]. The supernatant was then dried through freeze drying for two days. EPS produced was then determined gravimetrically and expressed in g/L.

### 4.9. Characterization of EPS

#### 4.9.1. Purification of EPS

The freeze-dried crude EPS was redissolved in deionized water and applied to a Sephacryl S-200 gel filtration column (2.0 cm × 60 cm) for further purification [46]. The sample was detected by UV wavelength detector and elution with deionized water at a flow rate of 0.6 mL/min. Dominating EPS fractions (4.8 mL/tube) were collected, combined, and lyophilized to obtain purified EPS.

#### 4.9.2. Molecular Mass Determination and Analysis of Monosaccharide Composition

The molecular weight of the EPS was determined on the SEC-MALLS-RI system, which consists of a pump (1525, Waters, Kitashinagawa, Japan), a sampler (High-Pressure Injection system, Wyatt Technology, Goleta, CA, USA) fitted with a 100 μL loop, a SEC column (Ultrahydrogel 250, 7.8 mm × 300 mm, Waters, Kitashinagawa, Japan), a MALLS detector (DAWN HELEOS II, Wyatt Technology, Goleta, CA, USA)and a refractive index detector (2414, Waters, Kitashinagawa, Japan). The solvent was 0.15 M aqueous NaCl (containing 0.02% NaN_3_). It was filtered through a 0.22 μm membrane and degassed before loading. The flow rate was set at 0.5 mL/min and 0.138 mL/g was selected as the value of refractive index increment (dn/dc) in 0.15 M aqueous NaCl solution at room temperature [47]. The column temperature was maintained at 25°C. An amount of 1000 μL of sample solution was prepared directly in 0.15 M NaCl at a concentration of 3 mg/mL and then purified by a 0.22 μm syringe filter before injection into the SEC-MALLS-RI system. The injection volume was 300 μL for each test in triplicate. Data acquisition and further analysis were conducted with the ASTRA software (Version 6.0, Wyatt Technology, Goleta, CA, USA) [48].

The monosaccharide composition of the purified EPS was determined by the 1-phenyl-3-methyl-5-pyrazolone high performance liquid chromatography (PMP-HPLC) method. The EPS purified fraction (2 mg) was hydrolyzed with 4 M trifluoroacetic acid (TFA) at 110 °C for 8 h in sealed glass tube. After cooling to room temperature, the residual acid was stripped by evaporation with nitrogen and the reaction solution was adjusted to pH 7.0 with 2 M NaOH, and then with 0.3 M NaOH. The hydrolysate was derivatized with 50 μL of 0.3 M NaOH and 50 μL of 0.5 M PMP solution at 70 °C for 100 min. We used chloroform to extract the hydrolysate and it was analyzed by HPLC with a ZORBAX Eclips eXDB-C18 column (Agilent, 5 μm, 4.6 mm × 250 mm, Santa Clara, CA, USA). The mobile phase A was aqueous containing sodium phosphate buffer (0.05 M, pH 6.9) and acetonitrile (vol; 85:15) and the mobile phase B was aqueous containing sodium phosphate buffer (0.05 M, pH 6.9) and acetonitrile (vol; 60:40). The time program of HPLC analysis was 0→10→30 min and the concentration program was 0%→8%→20% of the mobile phase B at a flow rate of 1 mL/min while the samples was detected by UV detection at 250 nm. The injection volume was 200 μL. The following standard sugars were used: mannose (Man), rhamnose (Rha), glucuronic acid (GluA), galacturonic acid (GalA), galactose (Gal), glucose (Glu), xylose (Xyl), arabinose (Ara), and fucose (Fuc) [49,50,51].

#### 4.9.3. Determination of Total Sugar, Uronic Sugar, and Protein

Total sugar of purified EPS fraction was determined with the phenol–sulfuric acid method using glucose as standard [50]. The uronic sugar content of the purified EPS was quantified by the PMP-HPLC method mentioned in Section 4.9.2. The protein content in the fraction was measured by the Coomassie brilliant blue method (Bradford method) using bovine serum albumin as standard [50,52].

#### 4.9.4. DSC Measurements

Differential scanning calorimetry (DSC, ZCEC-130263F, Mettler Toledo, Greifensee, Switzerland) was adopted to measure the Thermal properties of the purified EPS. The sample (10 mg) was loaded into an aluminum pan and covered with a lid. Then the sample was heated from 20 to 500 °C at a rate of 10 °C/min under nitrogen purge [53].

#### 4.9.5. FT-IR Spectroscopy Analysis

The IR spectrum of purified EPS was determined using a Nicolet AVA TAR370 Fourier-transform infrared spectrophotometer (FTIR, Nicolet, Madison, WI, USA) in the frequency range of 4000–400 cm^−1^ at room temperature. The sample was ground with spectroscopic grade KBr powder at a ratio of 1:50 and then pressed into a 1 mm pellet prior to FT-IR measurement [24].

### 4.10. Experimental Design

#### 4.10.1. Fractional Factorial Designs (FFD)

Factorial design is useful in identifying the important nutrients and interactions between two or more nutrients in relatively less experiments as compared to the one-factor at a time technique. The number of experiments can be reduced by using FFD without loss of information about the main effects [54]. To identify the most important ingredients in the culture medium, a 6 fractional factorial design leading to 20 sets of experiments was used to verify the most significant factors affecting the production of polysaccharide. All the experiments were performed in triplicate. The variables were coded according to the following equation:

x_i_ = (X_i_ − X_0_)/X_i_

where x_i_ is the coded value of an independent variable, X_i_ is the real value of an independent variable, X_0_ is the real value of an independent variable at the center point, and X_i_ is the step change value. The range and the levels of the variables with both coded values and natural values investigated in this study are given in Table 2. The polysaccharide production was considered as the dependent variable or response (Y_i_). The response surface is hence represented by a sloping plane. The next experiment was then carried out along the path of steepest ascent. 

#### 4.10.2. Central Composite Design (CCD)

In order to describe the nature of the response surface in the optimum region, a central composite design with five coded levels was performed. For predicting the optimal point, a second order polynomial function was fitted to the experimental results. For two factors this equation is:

y = b_0_ + b_1_x_1_ + b_2_x_2_ + b_12_x_1_x_2_ + b_11_x_12_ + b_22_x_22_

where y was the response variable, b_0_, b_1_, b_2_, b_11_, b_12_, b_22_ were the regression coefficients variables, for intercept, linear, quadratic, and interaction terms, respectively, and x_1_ and x_2_ were independent variables. Data were analyzed using the response surface regression (RSREG) procedure (SAS Institute Inc., Cary, NC, USA) and x is the coded level of the independent variable.

## 5. Conclusions

In summary, the effect of fermentation medium compositions on EPS production by a newly identified *K. rosea* ZJUQH was studied with one-factor-one-level and response surface methodology design. Response surface methodology proved to be a powerful tool in optimizing the culture medium for *K. rosea* ZJUQH. As the experimental results clearly showed, the EPSs produced by *K. rosea* ZJUQH depended mainly on the concentrations of KCl and MgSO_4_, especially MgSO_4_. High concentration of KCl and MgSO_4_ showed the most significant effects on the increase of EPS. Through statistically designed optimization, the EPS production by *K. rosea* ZJUQH could be increased from an average of 7.61 g/L in the initial medium to an average of 72.01 g/L in the optimized one, which means an approximate 846.25% enhancement. In this study, the optimized liquid culture medium for producing EPS by a *K. rosea* ZJUQH was composed of 5.0 g/L casein hydrolysate, 10.0 g/L sodium citrate, 3.0 g/L yeast extract, 5.0 g/L potassium chloride, 5.0 g/L peptone, and 58.0 g/L magnesium sulfate, at pH 7.0. A validated experiment of the fermentation process was also conducted in the 5.-L bioreactor. Further investigation into the intracellular substances as well as the structure and applications of the EPSs needs to be conducted in future study.

## Figures and Tables

**Table 1:** Formulae of five different fermentation culture media.

Number	Formula
M1	10 g/L peptone, 3 g/L beef extract, 3 g/L sodium citrate, 10 g/L MgSO_4_, 2 g/L KCl, 0.05 g/L FeSO_4_·7H_2_O, 5 g/L NaCl; pH 7.0–7.2.
M2	70g/L glucose, 10 g/L (NH_4_)_2_SO_4_, 1 g/L yeast extract, 3 g/L KH_2_PO_4_, 9 g/L K_2_HPO_4_, 0.4 g/L MgSO_4_·7H_2_O, 0.01 g/L MnSO_4_·H_2_O, 30 g/L NaCl; pH 7.0–7.2.
M3	5 g/L beef extract, 10 g/L peptone, 5 g/L KCl, 2.5 g/L MgSO_4_·7H_2_O; pH 7.0–7.2.
M4	20 g/L soluble starch, 0.5 g/L NaCl, 1 g/L KNO_3_, 0.5 g/L KH_2_PO_4_·3H_2_O, 2.5 g/L MgSO_4_·7H_2_O, 0.01 g/L FeSO_4_·7H_2_O; pH 7.4–7.6.
M5	5 g/L casein hydrolysate, 10 g/L sodium citrate, 3 g/L yeast extract, 2 g/L KCl, 5 g/L peptone, 10g/L MgSO_4_; pH 7.0–7.2.

**Table 2:** Range of values for Fractional Factorial Designs (FFD).

Independent Variables	Variable Name	Levels ^a^		
		−1	0	1
X_1_ (g/L)	Casein hydrolysate	2	5	8
X_2_ (g/L)	Sodium citrate	5	10	15
X_3_ (g/L)	Yeast extract	1	3	5
X_4_ (g/L)	KCl	1	2	3
X_5_ (g/L)	Peptone	2	5	8
X_6_ (g/L)	MgSO_4_	20	30	40

^a^ x_1_ = (X_1_ − 5)/3; x_2_ = (X_2_ − 10)/5; x_3_ = (X_3_ − 3)/2; x_4_ = (X_4_ − 2)/1; x_5_ = (X_5_ − 5)/3; x_6_ = (X_6_ − 30)/10.

**Table 3:** Experimental designs and the results of the FFD.

Run	x_1_	x_2_	x_3_	x_4_	x_5_	x_6_	Y (EPS, g/L)
1	1	−1	1	−1	−1	1	35.97
2	1	−1	−1	1	1	1	40.20
3	0	0	0	0	0	0	27.36
4	−1	−1	−1	−1	−1	−1	15.16
5	−1	1	1	−1	−1	−1	16.62
6	−1	1	1	1	−1	1	38.21
7	1	1	−1	1	−1	−1	20.40
8	−1	−1	1	1	1	−1	17.87
9	1	−1	−1	−1	1	−1	12.34
10	1	1	−1	−1	−1	1	36.88
11	−1	−1	−1	1	−1	1	38.87
12	−1	−1	1	−1	1	1	36.18
13	0	0	0	0	0	0	26.21
14	−1	1	−1	1	1	−1	27.25
15	0	0	0	0	0	0	27.42
16	−1	1	−1	−1	1	1	26.54
17	1	1	1	−1	1	−1	20.60
18	1	−1	1	1	−1	−1	19.28
19	0	0	0	0	0	0	30.41
20	1	1	1	1	1	1	44.77

**Table 4:** Results of the FFD regression analysis for exopolysaccharide (EPS) production (Y).

Source	Coefficient Estimate	Mean Square	*F* Value	Prob > F
Model	27.9463	126.9735	11.9082	0.0064
X_1_	0.8588	11.7992	1.1066	0.3410
X_2_	0.9625	14.8225	1.3901	0.2914
X_3_	0.7413	8.7912	0.8245	0.4055
X_4_	2.9100	135.4896	12.7069	0.0161
X_5_	0.2725	1.1881	0.1114	0.7521
X_6_	9.2563	1370.8506	128.5655	<0.0001
X_1_ × X_2_	0.8950	12.8164	1.2020	0.3229
X_1_ × X_3_	0.6088	5.9292	0.5561	0.4894
X_1_ × X_4_	−0.5525	4.8841	0.4581	0.5286
X_1_ × X_5_	0.4000	2.5600	0.2401	0.6449
X_1_ × X_6_	1.3938	31.0806	2.9149	0.1485
X_2_ × X_4_	0.8388	11.2560	1.0556	0.3513
X_2_ × X_6_	−1.5650	39.1876	3.6752	0.1134
Curvature		0.4234	0.0397	0.8499
Residual		10.6627		
Lack of Fit		22.2676	7.6101	0.0668
Pure error		2.9261		
Cor total				

**Table 5:** The steepest ascent design for EPSs formation by *K. rosea* ZJUQH.

Run	X_4_ (KCl)	X_6_ (MgSO_4_)	EPSs (g/L)
1	2.0	30	36.27
2	2.5	35	40.70
3	3.0	40	51.96
4	3.5	45	58.05
5	4.0	50	63.13
6	4.5	55	58.03

**Table 6:** Experimental design and results of the central composite design.

Run	x_4_	x_6_	Observed EPS ^a^ (g/L)	Predicted EPS (g/L)
1	−1	−1	52.56	52.47
2	1	1	48.01	50.39
3	0	1.414	75.91	71.77
4	0	−1.414	76.81	75.14
5	1	−1	62.94	65.56
6	0	0	67.34	66.47
7	0	0	45.29	43.30
8	−1.414	0	70.71	74.45
9	1.414	0	61.6	63.05
10	0	0	65.38	63.05
11	0	0	63.87	63.05
12	−1	1	60.53	63.05
13	0	0	63.86	63.05

^a^ x_4_ = (X_4_ − 4)/1; x_6_ = (X_6_ − 50)/8. Mean ± standard deviation (*n* = 3).

**Table 7:** ANOVA data of the central composite design *.

Source	Sum of Squares	DF	Mean Square	*F* Value	Prob > F
Model	1030.53	5	206.11	21.75	0.0004
x_4_	0.83	1	0.83	0.09	0.7762
x_6_	970.19	1	970.19	102.37	<0.0001
x_42_	15.32	1	15.32	1.62	0.2442
x_62_	30.27	1	30.27	3.19	0.1171
x_4_ × x_6_	7.43	1	7.43	0.78	0.4055
Residual	66.34	7	9.48		
Lack of fit	51.13	3	17.04	4.48	0.0907
Pure error	15.21	4	3.80		
Cor total	1096.88	12			

* R^2^ = 0.94 (*n* = 3).

## References

[1] Castillo-Carvajal L.C., Sanz-Martin J.L., Barragan-Huerta B.E. (2014). Biodegradation of organic pollutants in saline wastewater by halophilic microorganisms: A review. Environ. Scie. Pollut. Res. Int..

[2] Jindal N., Singh D.P., Khattar J.I.S. (2011). Kinetics and physico-chemical characterization of exopolysaccharides produced by the cyanobacterium *Oscillatoria formosa*. World J. Microbiol. Biotechnol..

[3] Sardari R.R.R., Kulcinskaja E., Ron E.Y.C., Björnsdóttir S., Friðjónsson Ó.H., Hreggviðsson G.Ó., Karlsson E.N. (2017). Evaluation of the production of exopolysaccharides by two strains of the thermophilic bacterium *Rhodothermus marinus*. Carbohydr. Polym..

[4] De Vuyst L., De Vin F., Vaningelgem F., Degeest B. (2001). Recent developments in the biosynthesis and applications of heteropolysaccharides from lactic acid bacteria. Int. Dairy J..

[5] Laws A., Gu Y., Marshall V. (2001). Biosynthesis, characterisation, and design of bacterial exopolysaccharides from lactic acid bacteria. Biotechnol. Adv..

[6] De Vuyst L., Vanderveken F., Van de Ven S., Degeest B. (1998). Production by and isolation of exopolysaccharides from *Streptococcus thermophilus* grown in a milk medium and evidence for their growth-associated biosynthesis. J. Appl. Microbiol..

[7] Mata J.A., Béjar V., Llamas I., Arias S., Bressollier P., Tallon R., Urdaci M.C., Quesada E. (2006). Exopolysaccharides produced by the recently described halophilic bacteria *Halomonas ventosae* and *Halomonas anticariensis*. Res. Microbiol..

[8] Tallon R., Bressollier P., Urdaci M.C. (2003). Isolation and characterization of two exopolysaccharides produced by *Lactobacillus plantarum* EP56. Res. Microbiol..

[9] Gandhi H.P., Ray R.M., Patel R.M. (1997). Exopolymer production by *Bacillus* species. Carbohydr. Polym..

[10] Nikinmaa M., Alam S.A., Raulio M., Katina K., Kajala I., Nordlund E., Sozer N. (2017). Bioprocessing of bran with exopolysaccharide producing microorganisms as a tool to improve expansion and textural properties of extruded cereal foams with high dietary fibre content. LWT Food Sci. Technol..

[11] Yamada T., Ogamo A., Saito T., Watanabe J., Uchiyama H., Nakagawa Y. (1997). Preparation and anti-HIV activity of low-molecular-weight carrageenans and their sulfated derivatives. Carbohydr. Polym..

[12] Sutherland I.W. (1998). Novel and established applications of microbial polysaccharides. Trends Biotechnol..

[13] DeAngelis P.L. (2012). Glycosaminoglycan polysaccharide biosynthesis and production: Today and tomorrow. Appl. Microbiol. Biotechnol..

[14] Sathiyanarayanan G., Seghal Kiran G., Selvin J. (2013). Synthesis of silver nanoparticles by polysaccharide bioflocculant produced from marine *Bacillus subtilis* MSBN17. Colloids Surf. B Biointerfaces.

[15] Mohamad O.A., Hao X., Xie P., Hatab S., Lin Y., Wei G. (2012). Biosorption of Copper (II) from Aqueous Solution Using Non-Living *Mesorhizobium amorphae* Strain CCNWGS0123. Microbes Environ..

[16] Wang Y., Ahmed Z., Feng W., Li C., Song S. (2008). Physicochemical properties of exopolysaccharide produced by *Lactobacillus kefiranofaciens* ZW3 isolated from Tibet kefir. Int. J. Biol. Macromol..

[17] Shah A.A., Hasan F., Hameed A., Ahmed S. (2008). Biological degradation of plastics: A comprehensive review. Biotechnol. Adv..

[18] Oren A., Gurevich P., Azachi M., Henis Y. (1992). Microbial degradation of pollutants at high salt concentrations. Biodegradation.

[19] Zhuang X.L., Han Z., Bai Z.H., Zhuang G.Q., Shim H.J. (2010). Progress in decontamination by halophilic microorganisms in saline wastewater and soil. Environ. Pollut..

[20] Joyce A.P., Leung S.S. (2013). Use of response surface methods and path of steepest ascent to optimize ligand-binding assay sensitivity. J. Immunol. Methods.

[21] Podell S., Emerson J.B., Jones C.M., Ugalde J.A., Welch S., Heidelberg K.B., Banfield J.F., Allen E.E. (2014). Seasonal fluctuations in ionic concentrations drive microbial succession in a hypersaline lake community. ISME J..

[22] Oren A. (2013). Life at high salt concentrations, intracellular KCl concentrations, and acidic proteomes. Front. Microbiol..

[23] Wu C.-S. (2009). Renewable resource-based composites of recycled natural fibers and maleated polylactide bioplastic: Characterization and biodegradability. Polym. Degrad. Stab..

[24] Lin L., Xie J., Liu S., Shen M., Tang W., Xie M. (2017). Polysaccharide from *Mesona chinensis*: Extraction optimization, physicochemical characterizations and antioxidant activities. Int. J. Biol. Macromol..

[25] Luo Q.-L., Tang Z.-H., Zhang X.-F., Zhong Y.-H., Yao S.-Z., Wang L.-S., Lin C.-W., Luo X. (2016). Chemical properties and antioxidant activity of a water-soluble polysaccharide from *Dendrobium officinale*. Int. J. Biol. Macromol..

[26] Ma Y., Galinski E.A., Grant W.D., Oren A., Ventosa A. (2010). Halophiles 2010: Life in saline environments. Appl. Environ. Microbiol..

[27] Oren A., Heldal M., Norland S., Galinski E.A. (2002). Intracellular ion and organic solute concentrations of the extremely halophilic bacterium *Salinibacter ruber*. Extrem. Life Extrem. Cond..

[28] Epstein W. (2003). The roles and regulation of potassium in bacteria. Prog. Nucleic Acid Res. Mol. Biol..

[29] Sleator R.D., Hill C. (2002). Bacterial osmoadaptation: The role of osmolytes in bacterial stress and virulence. FEMS Microbiol. Rev..

[30] Pastor J.M., Bernal V., Salvador M., Argandona M., Vargas C., Csonka L., Sevilla A., Iborra J.L., Nieto J.J., Canovas M. (2013). Role of Central Metabolism in the Osmoadaptation of the Halophilic Bacterium *Chromohalobacter salexigens*. J. Biol. Chem..

[31] Doan V.-T., Guzman H., Mai T.-H., Hatti-Kaul R. (2010). Ectoine Production by Halomonas boliviensis: Optimization Using Response Surface Methodology. Mar. Biotechnol..

[32] Tao P., Li H., Yu Y., Gu J., Liu Y. (2016). Ectoine and 5-hydroxyectoine accumulation in the halophile *Virgibacillus halodenitrificans* PDB-F2 in response to salt stress. Appl. Microbiol. Biotechnol..

[33] Amjres H., Béjar V., Quesada E., Carranza D., Abrini J., Sinquin C., Ratiskol J., Colliec-Jouault S., Llamas I. (2015). Characterization of haloglycan, an exopolysaccharide produced by *Halomonas stenophila* HK30. Int. J. Biol. Macromol..

[34] Palaniraj A., Jayaraman V. (2011). Production, recovery and applications of xanthan gum by *Xanthomonas campestris*. J. Food Eng..

[35] Shi Z., Zhang Y., Phillips G.O., Yang G. (2014). Utilization of bacterial cellulose in food. Food Hydrocoll..

[36] Yu L., Xu X., Zhou J., Lv G., Chen J. (2017). Chain conformation and rheological behavior of exopolysaccharide from *Bacillus mucilaginosus* SM-01. Food Hydrocoll..

[37] Chang C., Zhang L. (2011). Cellulose-based hydrogels: Present status and application prospects. Carbohydr. Polym..

[38] Rasulov B., Rozi P., Pattaeva M., Yili A., Aisa H. (2016). Exopolysaccharide-Based Bioflocculant Matrix of *Azotobacter chroococcum* XU1 for Synthesis of AgCl Nanoparticles and Its Application as a Novel Biocidal Nanobiomaterial. Materials.

[39] Chen T., Xu P., Zong S., Wang Y., Su N., Ye M. (2017). Purification, structural features, antioxidant and moisture-preserving activities of an exopolysaccharide from *Lachnum* YM262. Bioorg. Med. Chem. Lett..

[40] Morris E.R., Nishinari K., Rinaudo M. (2012). Gelation of gellan—A review. Food Hydrocoll..

[41] Kaur V., Bera M.B., Panesar P.S., Kumar H., Kennedy J.F. (2014). Welan gum: Microbial production, characterization, and applications. Int. J. Biol. Macromol..

[42] Xiao R., Zheng Y. (2016). Overview of microalgal extracellular polymeric substances (EPS) and their applications. Biotechnol. Adv..

[43] Cerning J. (1990). Exocellular polysaccharides produced by lactic-acid bacteria. FEMS Microbiol. Lett..

[44] Looijesteijn P.J., Boels I.C., Kleerebezem M., Hugenholtz J. (1999). Regulation of exopolysaccharide production by *Lactococcus lactis* subsp. cremoris by the sugar source. Appl. Environ. Microbiol..

[45] He P., Geng L., Wang Z., Mao D., Wang J., Xu C. (2012). Fermentation optimization, characterization and bioactivity of exopolysaccharides from *Funalia trogii*. Carbohydr. Polym..

[46] Zohra R.R., Aman A., Ansari A., Haider M.S., Qader S.A.U. (2015). Purification, characterization and end product analysis of dextran degrading endodextranase from *Bacillus licheniformis* KIBGE-IB25. Int. J. Biol. Macromol..

[47] Bednar B., Hennessey J.P. (1993). Molecular size analysis of capsular polysaccharide preparations from Streptococcus pneumoniae. Carbohydr. Res..

[48] Wei C.-Y., Li W.-Q., Shao S.-S., He L., Cheng J., Han S., Liu Y. (2016). Structure and chain conformation of a neutral intracellular heteropolysaccharide from mycelium of *Paecilomyces cicadae*. Carbohydr. Polym..

[49] Luo D. (2008). Identification of structure and antioxidant activity of a fraction of polysaccharide purified from *Dioscorea nipponica* Makino. Carbohydr. Polym..

[50] Xu R., Shen Q., Ding X., Gao W., Li P. (2011). Chemical characterization and antioxidant activity of an exopolysaccharide fraction isolated from *Bifidobacterium animalis* RH. Eur. Food Res. Technol..

[51] Li J.-H., Li S., Zhi Z.-J., Yan L.-F., Ye X.-Q., Ding T., Yan L., Linhardt R.J., Chen S.-G. (2016). Depolymerization of Fucosylated Chondroitin Sulfate with a Modified Fenton-System and Anticoagulant Activity of the Resulting Fragments. Mar. Drugs.

[52] Bradford M.M. (1976). Rapid and sensitive method for quantitation of microgram quantities of protein utilizing principle of protein-dye binding. Anal. Biochem..

[53] Lee I.W., Li J., Chen X., Park H.J. (2017). Fabrication of electrospun antioxidant nanofibers by rutin-pluronic solid dispersions for enhanced solubility. J. Appl. Polym. Sci..

[54] Li C., Bai J.H., Cai Z.L., Fan O.Y. (2002). Optimization of a cultural medium for bacteriocin production by *Lactococcus lactis* using response surface methodology. J. Biotechnol..

